# Circular RNA circCCNT2 is upregulated in the anterior cingulate cortex of individuals with bipolar disorder

**DOI:** 10.1038/s41398-021-01746-4

**Published:** 2021-12-10

**Authors:** Rixing Lin, Juan Pablo Lopez, Cristiana Cruceanu, Caroline Pierotti, Laura M. Fiori, Alessio Squassina, Caterina Chillotti, Christoph Dieterich, Nikolaos Mellios, Gustavo Turecki

**Affiliations:** 1 McGill Group for Suicide Studies, Douglas Mental Health University Institute, Department of Psychiatry, McGill University, Montreal, QC Canada; 2 Integrated Program in Neuroscience, McGill University, Montreal, QC Canada; 3 Department of Stress Neurobiology and Neurogenetics, Max Planck Institute of Psychiatry, Munich, Bavaria 80804 Germany; 4 Department of Translational Research in Psychiatry, Max Planck Institute of Psychiatry, 80804 Munich, Germany; 5 Department of Neurosciences, University of New Mexico School of Medicine, Albuquerque, NM USA; 6 Department of Biomedical Sciences, Section of Neuroscience and Clinical Pharmacology, University of Cagliari, Cagliari, Italy; 7 Unit of Clinical Pharmacology, University Hospital Agency of Cagliari, Cagliari, Italy; 8 Section of Bioinformatics and Systems Cardiology, Klaus Tschira Institute for Integrative Computational Cardiology, Heidelberg, Germany; 9 Department of Internal Medicine III (Cardiology, Angiology, and Pneumology), University Hospital Heidelberg, Heidelberg, Germany; 10 DZHK (German Centre for Cardiovascular Research), Partner Site Heidelberg/Mannheim, Berlin, Germany; 11 Autophagy inflammation and metabolism (AIM) center, Albuquerque, NM USA

**Keywords:** Molecular neuroscience, Bipolar disorder

## Abstract

Gene expression dysregulation in the brain has been associated with bipolar disorder, but little is known about the role of non-coding RNAs. Circular RNAs are a novel class of long noncoding RNAs that have recently been shown to be important in brain development and function. However, their potential role in psychiatric disorders, including bipolar disorder, has not been well investigated. In this study, we profiled circular RNAs in the brain tissue of individuals with bipolar disorder. Total RNA sequencing was initially performed in samples from the anterior cingulate cortex of a cohort comprised of individuals with bipolar disorder (*N* = 13) and neurotypical controls (*N* = 13) and circular RNAs were identified and analyzed using “circtools”. Significant circular RNAs were validated by RT-qPCR and replicated in the anterior cingulate cortex in an independent cohort (24 bipolar disorder cases and 27 controls). In addition, we conducted in vitro studies using B-lymphoblastoid cells collected from bipolar cases (*N* = 19) and healthy controls (*N* = 12) to investigate how circular RNAs respond following lithium treatment. In the discovery RNA sequencing analysis, 26 circular RNAs were significantly differentially expressed between bipolar disorder cases and controls (FDR < 0.1). Of these, circCCNT2 was RT-qPCR validated showing significant upregulation in bipolar disorder (*p* = 0.03). This upregulation in bipolar disorder was replicated in an independent post-mortem human anterior cingulate cortex cohort and in B-lymphoblastoid cell culture. Furthermore, circCCNT2 expression was reduced in response to lithium treatment in vitro. Together, our study is the first to associate circCCNT2 to bipolar disorder and lithium treatment.

## Introduction

Bipolar disorder (BD) is a debilitating and chronic psychiatric disorder characterized by cyclic mood episodes, including mania, hypomania, and depression [1]. BD affects approximately 1% of the general population and increases the risk of suicide [2–4]. It is unlikely that a single etiological factor is associated with BD as several environmental, genetic, epigenetic, and transcriptomic factors appear to play important roles in the BD pathology. While there has been some previous work investigating the role of non-coding RNAs in BD, circular RNAs (circRNAs), an intriguing new class of noncoding RNAs, have remained understudied [5, 6].

Circularized RNAs were first observed by electron microscopy in the early 1980s [7–9]. With the development of RNA sequencing, researchers observed strange scrambled exon orders starting in the early 1990s [10–18]. Thus began the infancy of circRNA research, however, it was not until the implementation of massive parallel sequencing and modern computation capabilities that circRNAs were properly characterized [19–21]. CircRNAs are a category of long noncoding RNAs and make up a diverse and abundant collection of circularized RNA transcripts formed through a process known as back-splicing, where linear RNA loops in on itself and the 5′ and 3′ ends are covalently joined [22, 23]. CircRNAs are generally composed of one to five exons primarily arising from protein-coding genes; however, intronic regions can also be incorporated into the circularized transcript [24, 25]. Interestingly, circRNAs are temporally regulated and show tissue-specific expression patterns, with enrichment in brain tissue, making them an exceptionally interesting category of RNA to investigate in the context of psychiatric disorders [26–28]. While their function is not yet fully understood, some circRNAs have shown the ability to sponge microRNAs or RNA-binding proteins (RBPs)/transcription factors by containing complementary microRNA sequences or sequence motifs for protein binding [29–33]. That said, much has still to be discovered on the function of the large majority of circRNAs as there is no unifying mechanism of action which applies to all circRNAs.

Recent work has shown that circRNA dysregulation is associated with psychiatric disorders, making them an exciting new avenue of investigation for molecular psychiatry research [31, 33–35]. Using RNA sequencing followed by bioinformatic identification of back-spliced reads allows a non-biased approach to identify and profile global circRNA expression. In this study, we identified differential expression of circRNAs between individuals with BD and psychiatrically healthy controls in post-mortem human anterior cingulate cortex (ACC) (Brodmann area 24). Differentially expressed circRNAs from RNA sequencing were validated and further interrogated in an external replication cohort followed by an investigation in vitro.

## Methods and materials

### Human post-mortem ACC

#### Discovery cohort

Post-mortem human brain samples from ACC were obtained from the Douglas-Bell Canada Brain Bank (Douglas Mental Health University Institute, Montreal, Quebec, Canada) [5]. All individuals included in this study were of French–Canadian origin and psychological autopsies were performed, as previously described by Dumais et al. [36], on both cases and controls using clinical information ascertained by structured interviews and interpreted using best-consensus methods by a panel of clinicians in order to elicit diagnoses based on DSM-IV criteria. A total of 26 brain samples (13 individuals with BD and 13 psychiatrically healthy controls) were included in this study. Cases included individuals who had a diagnosis of BD type I or type II. The control group had no history of psychopathology, including suicidal behavior, major mood or psychotic disorders; subjects in the control group died suddenly by accidental causes or myocardial infarction. This study was approved by the Douglas Hospital Research Ethics Board, and written informed consent was obtained from the next-of-kin for each subject through an agreement with the Quebec Coroner’s Office.

##### Library Preparation and RNA sequencing

Library preparation and RNA sequencing were conducted as described by Cruceanu et al. [5]. Briefly, total RNA was extracted from brain tissue using the RNeasy kit (Qiagen). Ribosome depleted RNA was used to construct libraries using TruSeq dUTP degradation-based directional protocol (Illumina). All sequencing was carried out at the Genome Quebec Innovation Center using the Illumina HiSeq 2000 platform.

##### Bioinformatics analysis

Illumina RNA-seq reads were pre-processed with Flexbar 3 for quality clipping and sequencing adapter removal [37]. We used Bowtie2 and human rRNA reference transcripts to subtract rRNA reads in silico [38]. All remaining reads were aligned against the human genome using STAR (2.5.3), guided by the EnsEMBL v90 reference annotation [39]. Back-splice junctions were reported in the Chimeric.out.junction file. We identified circular RNAs by our software circtools [40]. Briefly, we employed the detect and circtest modules to identify circular RNAs and to quantify circular RNAs relative to their host gene expression.

##### SYBR Green RT-qPCR Validation

Total RNA was reverse transcribed to cDNA using M-MLV reverse transcriptase (Gibco) following the manufacturer’s protocol with random hexamers. Real-time quantitative PCR (RT-qPCR) reactions were run in triplicates using the QuantStudio 6 Flex Real-Time PCR System and data were collected using the QuantStudio Real-Time PCR Software v1.1 (Applied Biosystems). To measure RNA expression, we used custom-designed probes (Supplementary Table 1) with PowerUp SYBR Green Master Mix (ThermoFisher). Expression levels were calculated using the absolute quantitation standard curve method, with GAPDH used as endogenous control.

#### Replication cohort

Post-mortem human ACC samples were obtained from the Stanley Medical Research Institute as described by Zimmerman et al. [34]. A detailed description of inclusion/exclusion criteria for donors with BD and controls can be found at Torrey et al. [41]. Here, we used a subset of the cohort described by Zimmerman et al. [20], which includes a total of 51 brain samples (24 individuals with BD and 27 psychiatrically healthy controls).

##### TaqMan RT-qPCR quantification

Reverse transcription was performed using the SuperScript IV First-Strand Synthesis System (ThermoFisher Scientific) with random hexamers. cDNA was then used together with a custom TaqMan probe (Supplementary Table 1), which spans the back-splice junction of circCCNT2, and TaqMan Universal PCR Master Mix (ThermoFisher Scientific) for RT-qPCR. RT-qPCR quantification was performed using the following formula: relative value = 2^CtcircTulp4^/2^CtcircCCNT2^; circTulp4 was used for normalization.

### Circularization confirmation

CircCCNT2 primers were run on an agarose gel and were sequence-validated. Moreover, resistance to RNase R and reduced abundance in oligo-dT reverse-transcribed cDNA was tested.

#### Sanger sequencing

Approximately, 50 ng of cDNA was PCR amplified (95 °C for 10 min; 40 cycles of 95 °C for 15 s and 60 °C for 1 min) using custom-designed primers for circCCNT2 followed by gel electrophoresis for product purification. The expected band product (180nt) was cutout for gel purification (NEB gel purification kit). Sequencing was performed at Genome Quebec Innovation Center on a 3730xl DNA analyzer platform (Applied Biosystem). The same forward and reverse primers used for qPCR were used for Sanger sequencing (Supplementary Table 1).

#### RNase R Treatment

Totally, 2 µg of total RNA was treated with RNase R (20 U/µL; epicenter) following the manufacturer’s protocol in a 5uL reaction. Control RNA was treated the same with the absence of RNase R enzyme. RNase R and control samples were incubated at 37 °C for 3 h followed by phenol–chloroform pull down to purify the RNA. Each condition was split into two aliquots where one aliquot used oligonucleotide dT-based cDNA synthesis and the other used random hexamer-based cDNA synthesis (cDNA synthesis using M-MLV reverse transcriptase as described above).

### B lymphoblastoid cohort

B lymphoblastoid cell lines (B-LCL) were collected and immortalized from peripheral blood samples of BD patients and healthy controls as described by Squassina et al. [42]. In short, each of the 31 B-LCL samples (19 cases and 12 controls) was split into two equal aliquots and cultured in separate flasks; one with culture media containing 1 mM of lithium chloride (Li) and one without for seven days (Fig. 3A). B-LCLs were pelleted via centrifugation for total RNA extraction using miRNeasy Mini Kit (Qiagen) following the manufacturer’s protocol. Total RNA was reverse transcribed using M-MLV reverse transcriptase (Gibco) following the manufacturer’s protocol with random hexamer priming. To measure RNA expression, we used custom-designed probes (IDT) with PowerUp SYBR Green Master Mix (ThermoFisher) as described above. GAPDH and 18S rRNA was used for the normalization of circCCNT2.

### Prediction algorithms for circCCNT2 interactions and functional outcomes

Algorithms within circAtlas, circBank, and circular RNA Interactome were used to determine miRNA or RBPs that interact with circCCNT2 [43–45]. STRING v11 was used to determine protein interaction networks and functional enrichment analysis for circCCNT2 interacting RBPs [46]. miRwalk 2.0 was used to determine gene targets for circCCNT2 interacting miRNAs [47, 48]. Gene ontology enrichment analysis was used for predicted miRNA gene targets [49, 50]. Algorithms within circAtlas and circBank were used to identify open reading frames (ORFs) and internal ribosome entry site (IRE) elements in circCCNT2 [44, 45]. MEME suite was used for GRACH (R = G or C) sequence motif identification [51, 52].

### Statistical analysis

Statistical analyses were performed using IBM SPSS Statistics 22.0 and GraphPad Prism 9. Student two-tailed *t* tests were used to assess expression changes between BD and control. A two-way mixed ANOVA test was performed with Bonferroni correction to assess group, Li treatment, and group by Li treatment interaction effects for the B-LCL dataset.

## Results

### circRNAs associated with bipolar disorder in human post-mortem ACC

To explore the role of circRNAs in BD, we used RNA sequencing data previously generated by our laboratory from human post-mortem ACC samples comprising 13 individuals with BD and 13 age, brain pH, and RNA integrity number matched psychiatrically healthy controls [5].

**Fig. 1:**
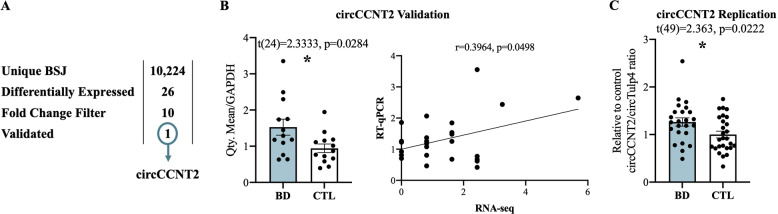
circCCNT2 expression in ACC of individuals with bipolar disorder. **A** Filtering of circRNA from sequence identified back splice junctions (BSJ), regulation in bipolar disorder, fold-change, and RT-qPCR validation. **B** circCCNT2 validation by RT-qPCR (left). circCCNT2 expression correlation between RNA sequencing and RT-qPCR (right). **C** Expression of circCCNT2 in an independent human postmortem ACC (BA24) replication cohort. Student’s two-tailed *t* tests were used to assess mean differences between individuals with bipolar disorder (BD) and controls (CTL). Pearson correlation coefficient (*r*) was used for correlation analysis. All bar plots represent the mean with individual data points as dots. Error bars represent S.E.M. (*<0.05).

Our RNA sequencing dataset identified over 10,000 unique circRNAs, of which 26 circRNAs were significantly differentially expressed between BD and controls after FDR < 0.1 corrections (Fig. 1A, Supplementary Table 2). The top ten most differentially regulated circRNAs, comprising the top five most upregulated and the top five most downregulated, were selected for further investigation (Table 1). We were interested to see if our candidate circRNAs were co-expressed with each other and thus may be involved in similar molecular mechanisms contributing to BD. Interestingly we observed a strong correlation of expression between all the top five most upregulated circRNAs whereas the top five most downregulated circRNAs did not show such consistent correlations (Supplementary Fig. 1). To confirm the differential expression of our ten candidate circRNAs we designed custom primers that specifically amplify the predicted back-splice junction for RT-qPCR validation. Of the circRNAs tested, circCCNT2 was the only transcript that reached statistical significance in our validation measurements, (*t*(24) = 2.276, *p* = 0.0320), and displayed a similar direction and level of upregulation to that observed in our RNA sequencing dataset (Fig. 1B, Supplementary Fig. 2, Supplementary Fig. 3). We next sought to replicate our findings in an independent human post-mortem ACC cohort [34]. To do so, we measured circCCNT2 expression using TaqMan-based RT-qPCR in 24 individuals with BD and 27 psychiatrically healthy controls. We again observed a significant upregulation of circCCNT2 in BD compared to controls (*t*(49) = 2.363, *p* = 0.0222) (Fig. 1C).

**Table 1:** Top ten differentially expressed circRNAs in BA24 of individuals with bipolar disorder^a^.

circRNA name^b^	CircRNA ID^c^	Host gene description	Genomic location^d^	Strand	BSJ^e^	Size^f^	*p*-Value	Adjusted *p* value^g^	Fold change (%)
circCCNT2	hsa_circ_0056537	Cyclin T2	chr2:134936841–134942674	+	exon5–exon3	253nt	0.00006	0.09035	83
circCLOCK	hsa_circ_0126631	Clock circadian regulator	chr4:55475963–55482828	−	exon8–exon5	391nt	0.00021	0.09362	82
circRERE	hsa_circ_0002158	Arginine–glutamic acid dipeptide repeats	chr1:8541214–8557523	−	exon8–exon6	308nt	0.00013	0.09035	66
circSGMS1	hsa_circ_0093713	Sphingomyelin synthase 1	chr10:50433476–50519921	−	exon6–exon3	357nt	0.0001	0.09035	43
circPTPN4	hsa_circ_0117151	Protein tyrosine phosphatase non-receptor type 4	chr2:119809837–119900806	+	exon10–exon2	781nt	0.00008	0.09035	36
circUBR5	N/A	Ubiquitin protein ligase E3 component N-recognin 5	chr8:102271134–102272772	−	exon49–exon48	356nt	0.00009	0.09035	−21
circGLRB	hsa_circ_0125612	Glycine receptor beta	chr4:157120556–157153010	+	exon9–exon3	1075nt	0.00003	0.09035	−38
circCYFIP2	hsa_circ_0074763	Cytoplasmic FMR1 interacting protein 2	chr5:157294783–157304366	+	exon7–exon4	588nt	0.00023	0.09362	−45
circSHC3	hsa_circ_0003708	SHC adaptor protein 3	chr9:89037993–89046994	−	exon11–exon8	694nt	0.00014	0.09035	−51
circLRBA	hsa_circ_0071174	LPS responsive beige-like anchor protein	chr4:150735258–150808398	−	exon36–exon32	449nt	0.00013	0.09035	−55

^a^CircRNA expression quantification followed by differential expression analysis and fold change filtering identified ten candidate circRNAs (BA: Brodmann’s Area).

^b^In this study circRNAs were named as their host gene with “circ” prefix.

^c^circRNA ID as listed in circBase. circUBR5 did not have a known circRNA ID and is believed to be a newly identified splice variant.

^d^Genomic location is outlined as follows: chromosome#:start-end for GRch38/hg38 build.

^e^BSJ: back splice junction. This column indicates between which two exons the back splice event occurred.

^f^Size of the mature circRNA splice variant in nucleotides (nt).

^g^Nominal *p*-values were adjusted for false discovery rate (FDR) correction; significant at an FDR of <0.1.

**Fig. 2:**
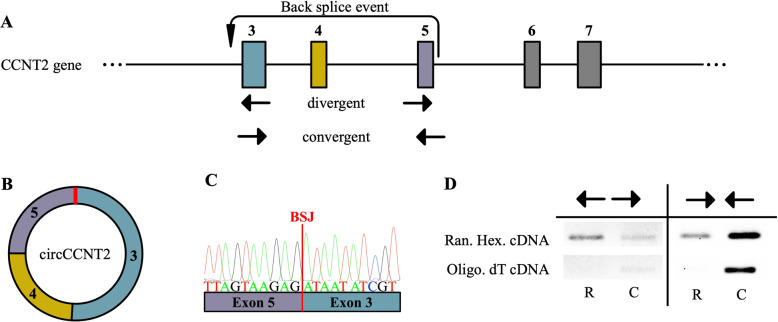
circCCNT2 circularization confirmation. **A** Schematic diagram of the predicted back splicing event that forms circCCNT2. Rectangles indicate exons (the number above indicates exon number), the solid line indicates introns, and the dotted line indicates a continuation of the CCNT2 gene up and downstream. Arrows below exon 3 and exon 5 indicate the location and direction of divergent and convergent primers as would appear on the linear CCNT2 gene. **B** Schematic diagram of the mature circCCNT2 transcript as a result of exon 5 to exon 3 back splice event. The red bar indicates the back splice junction. **C** Sanger sequencing confirming back splice junction sequence (BSJ back splice junction). **D** Divergent primer amplification showed enrichment of circCCNT2 in RNase R treated RNA compared to the non-treated control. Divergent primers failed to amplify circCCNT2 in oligonucleotide dT constructed cDNA since circRNAs do not contain a polyA tail. Convergent primer amplification of linear CCNT2 showed the opposite amplification profile as described for divergent primer amplification. Black arrows indicate divergent and convergent primers as described in (**A**) (Ran. Hex. random hexamer, Oligo. oligonucleotide, R RNase R treated RNA, C non-treated RNA).

The host gene for circCCNT2 encodes the cyclin T2 protein, which is a member of the highly conserved cyclin family whose expression is tightly related to the cell cycle [53]. Cyclin T2 is a regulator of the catalytic activity of CDK9 (cyclin-dependent kinase 9). Together, cyclin T2 and CDK9 comprise a subunit of the P-TEFb (positive transcription elongation factor b) multiprotein complex, which is involved in transcriptional regulation [53]. Furthermore, the CCNT2 gene is predicted to produce 12 specific circCCNT2 variants [54]. The specific variant of circCCNT2 we measured is composed of back-splice events from exon 5 to exon 3 of the CCNT2 gene (Fig. 2A, B) and is also referred to as hsa_circ_0056537. Here, we refer to this specific variant as circCCNT2.

To confirm the predicted back splice event and circular structure of circCCNT2, primers were sequence validated for amplification of the predicted back-splice junction, and an RNase R treatment assay was used to confirm the circular structure. Sanger sequencing confirmed that our primers were specifically amplifying the predicted back-splice junction between exon 3 and exon 5 of the CCNT2 gene (Fig. 2C). RNase R digests linear RNA 3′ to 5′, allowing circRNAs to evade digestion due to the absence of free 3′ end. Furthermore, circRNAs are depleted in oligo dT constructed cDNA due to the lack of a polyA tail. Expression of circCCNT2 was enriched in RNase R treated RNA, compared to non-treated control RNA, and depleted in oligo dT constructed cDNA (Fig. 2D). Together these experiments indicated that we specifically measured circCCNT2 with back-splice junction formed from exon 5 and exon 3 and confirmed its circular structure beyond bioinformatic prediction.

### circCCNT2 is altered in B-LCL cells of individuals with BD and by lithium treatment

**Fig. 3:**
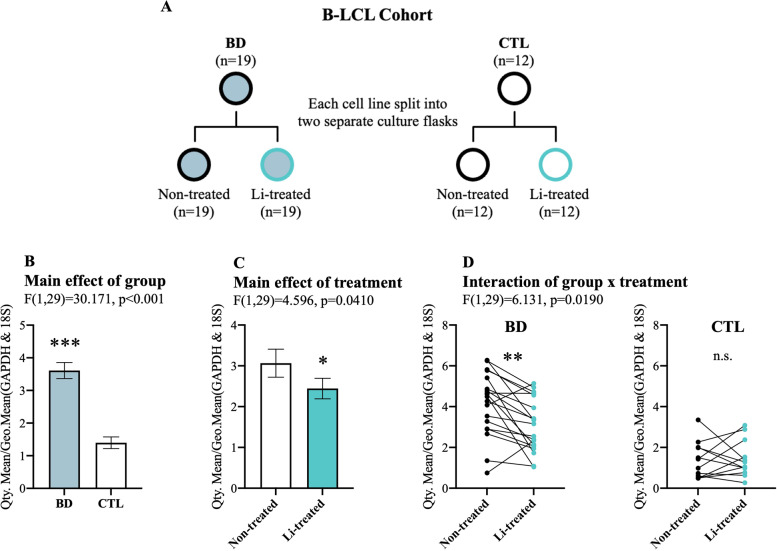
Replication and effects of lithium treatment on circCCNT2 expression. **A** Schematic diagram of the B-LCL cohort. **B** Expression of circCCNT2 in B-LCL cells collected from individuals with bipolar disorder (BD) and controls (CTL). Showing the main effect of the group from a two-way mixed ANOVA. **C** Expression of circCCNT2 in B-LCL cells that were treated and not treated with lithium. Showing the main effect of treatment from a two-way mixed ANOVA. **D** Expression of circCCNT2 showing the decomposition of the significant interaction of group by treatment from a two-way mixed ANOVA. Lithium treatment specifically reduces circCCNT2 expression in individuals with bipolar disorder with no effect in control subjects. All bar graphs represent the mean expression of circCCNT2; each dot represents a single data point; error bars represent SEM; *<0.05, **<0.01, ***<0.001, n.s. not significant.

Lithium treatment is a first-line prophylactic treatment for BD [55–57]. We next sought to see if lithium influences the expression of circCCNT2. B Lymphoblastoid cells (B-LCLs) were collected from 19 bipolar cases and 12 healthy controls. Each cell line was divided in half and treated with lithium or left untreated (Fig. 3A). CircCCNT2 expression showed a significant main effect of group (*F*(1, 29) = 30,171, *p* = 0.00000645), treatment (*F*(1, 29) = 4.596, *p* = 0.0410), and a significant interaction of group by treatment (*F*(1, 29) = 6.131, *p* = 0.0190) (Fig. 3B–D). Similar to our results in the brain, we observed an upregulation of circCCNT2 in individuals with BD (Fig. 3B). Interestingly, lithium treatment reversed the upregulation seen in the BD group and downregulated circCCNT2 expression (Fig. 3C). Furthermore, the expression of circCCNT2 was specifically reduced in BD B-LCLs treated with lithium (*F*(1, 18) = 11.740, *p* = 0.006), but not in control B-LCLs treated with lithium (*F*(1, 11) = 0.066, *p* = 1.000) (Fig. 3D). This indicates that the significant main effect of lithium treatment was predominantly driven by the BD group.

### Functional significance of circCCNT2

CircRNAs have functional biological significance and regulatory roles through interaction with miRNA, RNA-binding proteins (RBPs), or translation into peptides [32, 58, 59]. To assess circCCNT2’s ability to sequester RBPs we utilized circAtlas and found at least one binding site for 26 unique RBPs. Using STRING, we discovered these 26 RBPs are enriched for terms related to post-transcriptional regulation of both pre-mRNA and mRNA (Supplementary Fig. 4A and Supplementary Table 3). Using three different miRNA prediction platforms, miR-877-5p was consistently predicted to bind to circCCNT2 [43–45]. Gene ontology (GO) analysis of miR-877-5p targets showed cellular component terms related to synapse formation (Supplementary Table 4). Using the circCCNT2 sequence we did not identify any ORFs or presence of IRE elements indicating a low probability of protein-coding potential. However, we did observe an abundance of GRACH (R = G or C) sequence motifs which indicates the possibility of m6A modification on circCCNT2, which has shown to be associated with protein-coding potential in circRNAs (Supplementary Fig. 4B) [58, 59].

## Discussion

Despite the growing body of work focused on noncoding RNAs, very little is known about the role of circRNAs, particularly in psychiatric disorders, such as BD. Here, we showed that circCCNT2 is significantly upregulated in BD in three independent cohorts and two tissue types. Furthermore, circCCNT2 is downregulated in response to lithium treatment in vitro.

While very few studies have investigated circRNAs in BD, circNEBL and circEPHA3 were described as significantly upregulated in the medial frontal gyrus of individuals with bipolar disorder [60]. Moreover, in a different study, circHOMER1a and circADAM22 were significantly downregulated in the orbitofrontal cortex (OFC) of individuals with BD, findings that were validated by qPCR [34]. Interestingly, knocking down circHOMER1 in the mice OFC showed behaviors related to cognitive defects and alterations in transcriptomic profiles [34]. Although we were able to identify circNEBL, circEPHA3, circHOMer1, and circADAM22 in our ACC dataset, we were not able to replicate previous findings. However, these studies investigated different brain regions which could point to brain region-specific regulation of circRNAs in BD. Another study investigating peripheral samples of BD patients and healthy controls, identified 33 circRNAs as nominally differentially expressed between groups [61]. Our study was the first to point to circCCNT2 as possibly implicated in BD.

RBPs that are predicted to interact with circCCNT2 show enrichment for RNA regulatory proteins. This suggests that the altered circCCNT2 expression in BD could play a role in the regulation of differentially expressed genes associated with BD. Moreover, Cruceanu et al. [5] did not find CCNT2 to be differentially expressed between BD and controls, in the same cohort used in this study, giving evidence that circCCNT2 has specific roles in BD independent of its linear host gene counterpart. We also identified miR-877-5p binding sites on circCCNT2. Interestingly, miR-877-5p and miR-877-3p have been reported to interact with several other circRNAs in relation to cancer progression [62–64]. However, this relationship has not previously been reported in the context of any neuropsychiatric disorders. When we performed enrichment analysis on predicted targets of miR-877-5p, we observed several cellular component terms related to neuronal synapses. We also observed molecular function terms related to ion binding and hydrolase and biological processes terms such as neurogenesis and cell signaling. Together, this gives the possibility that the predicted circCCNT2/miR-877-5p interaction might regulate genes involved in modulating neuronal signaling in BD and lithium treatment and may also be involved in lithium metabolism. This possible interaction of circCCNT2 with RBPs and miRNAs may be an interesting avenue for future work in BD research.

Our study has many advantages including the use of RNA sequencing as a discovery method to profile global circRNA expression, the use of three independent cohorts and two tissue types to replicate our findings, and the use of multiple measuring techniques and other assays to confidently assess the levels of specific circularized RNAs. However, there are several limitations to this study. The sample sizes of our cohorts are relatively small, but we were able to identify circCCNT2 and replicate it in an independent sample. Although we are able to confirm the circular structure of cirCCNT2 and its association to BD, the function of circCCNT2 remains unknown. Much more detailed analyses exploring the potential mechanism of action for circCCNT2 are needed to understand its role in BD pathology and lithium response. Finally, the relationship between the expression of peripheral and central circRNAs is unclear. However, given the consistency of dysregulation between the peripheral and central tissues, it is possible that circRNAs may cross the blood–brain barrier. For example, it has been shown that circRNAs are packaged into exosomes, or other extracellular vesicles, which have been shown to cross the blood–brain barrier [65–71]. Altogether, our study is the first to demonstrate that circCCNT2 is associated with BD and lithium treatment and has predicted functional implications related to neuronal functioning.

## Supplementary information

Supplemental Material

Supplemental Table 2

Supplemental Table 3

Supplemental Table 4
