# Supplementary material for: Circular RNA circCCNT2 is upregulated in the anterior cingulate cortex of individuals with bipolar disorder
Source: Transl Psychiatry. 2021 Dec 10;11:629. doi: 10.1038/s41398-021-01746-4 (PMC8664854; doi:10.1038/s41398-021-01746-4)
Supplement: Supplementary file 1 — Supplemental Material [file 41398_2021_1746_MOESM1_ESM.docx]

**SUPPLEMENTAL MATERIAL**

**Circular RNA circCCNT2 is upregulated in the anterior cingulate cortex of individuals with bipolar disorder**

Rixing Lin, Juan Pablo Lopez, Cristiana Cruceanu, Caroline Pierotti, Laura M. Fiori, Alessio Squassina, Caterina Chillotti, Christoph Dieterich, Nikolaos Mellios, Gustavo Turecki

**Supplementary Figure 1.** **Correlation matrix of the top 10 circRNAs.**

Legend corresponds to r value; *p<0.05

**Supplementary Figure 2. circRNA validation by RT-qPCR (related to Table 1 and Figure1)**

**A-I** CircRNA expression as measured by RT-qPCR with custom designed probes that amplify the predicted back splice junction. Student’s t-tests were used to assess significance between individuals with bipolar disorder (BD) and controls (CTL). All bar plots represent the mean with individual data points as dots. Error bars represent SEM (*<0.05).

**Supplementary Figure 3. circRNA validation correlation with RNA sequencing (related to Table 1 and Figure1)**

**A-I** Correlation between circRNA expression from RNA-seq discovery and RT-qPCR validation. Pearson correlation coefficient (r) and significance level (p) are reported beneath each circRNA name.

**Supplementary Figure 4. circCCNT2 Functional Predictions**

**A** Protein-Protein interaction of the 26 RBPs predicted to interact with circCCNT2. Proteins excluded were not a part of any interaction. **B** GRACH motif was significantly enriched within the mature circCCNT2 transcript sequence.

**Supplementary Table 1. Primer Sequences**

**Supplementary Table 2. circRNAs identified from RNA sequencing (related to table 1)**

See Excel document

**Supplementary Tables 3. circCCNT2 RBP functional enrichment analysis**

See Excel document

**Supplementary Tables 4. miR877-5p target gene ontology analysis**

See Excel document

**Supplementary Figure 1**

**
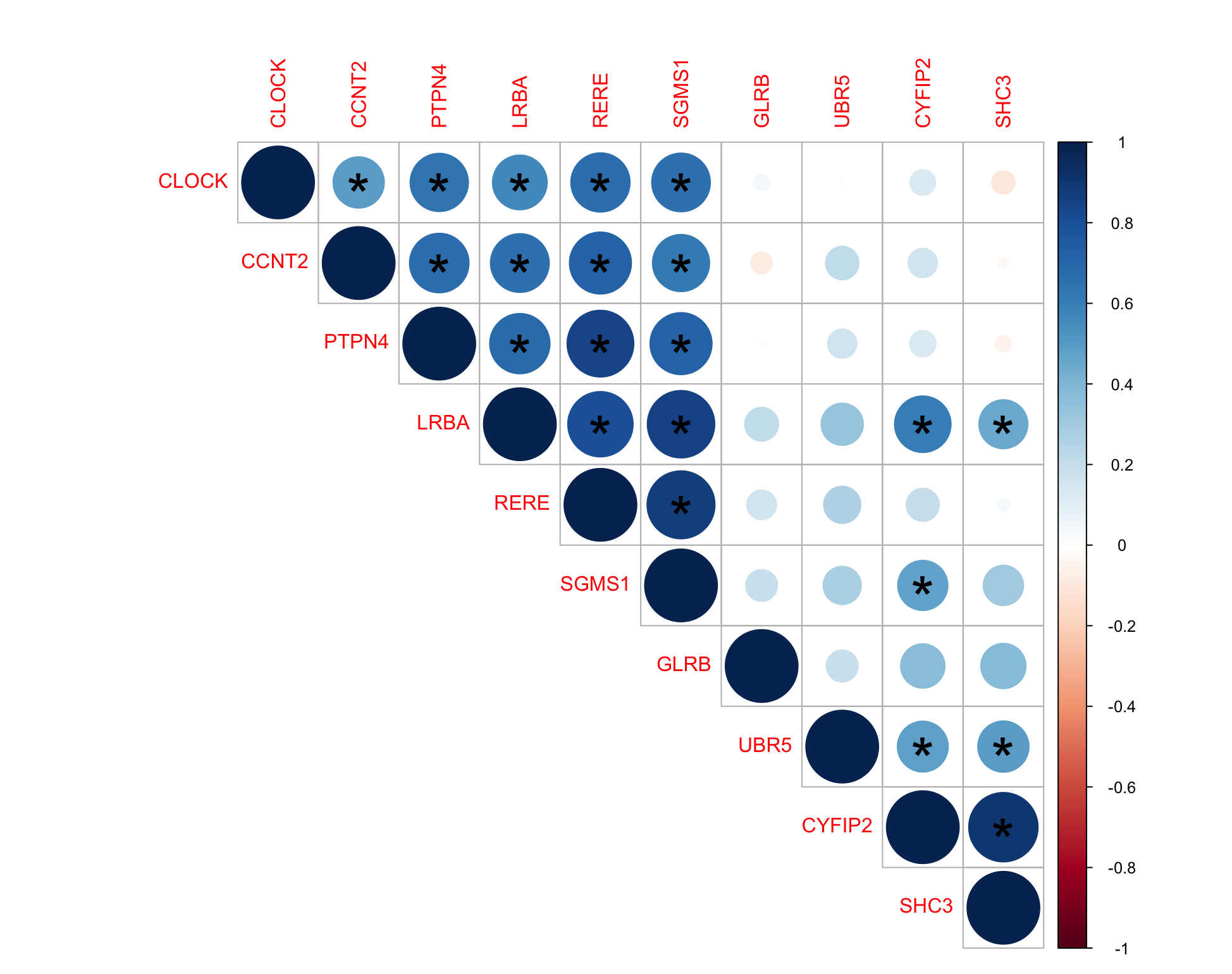
**

**
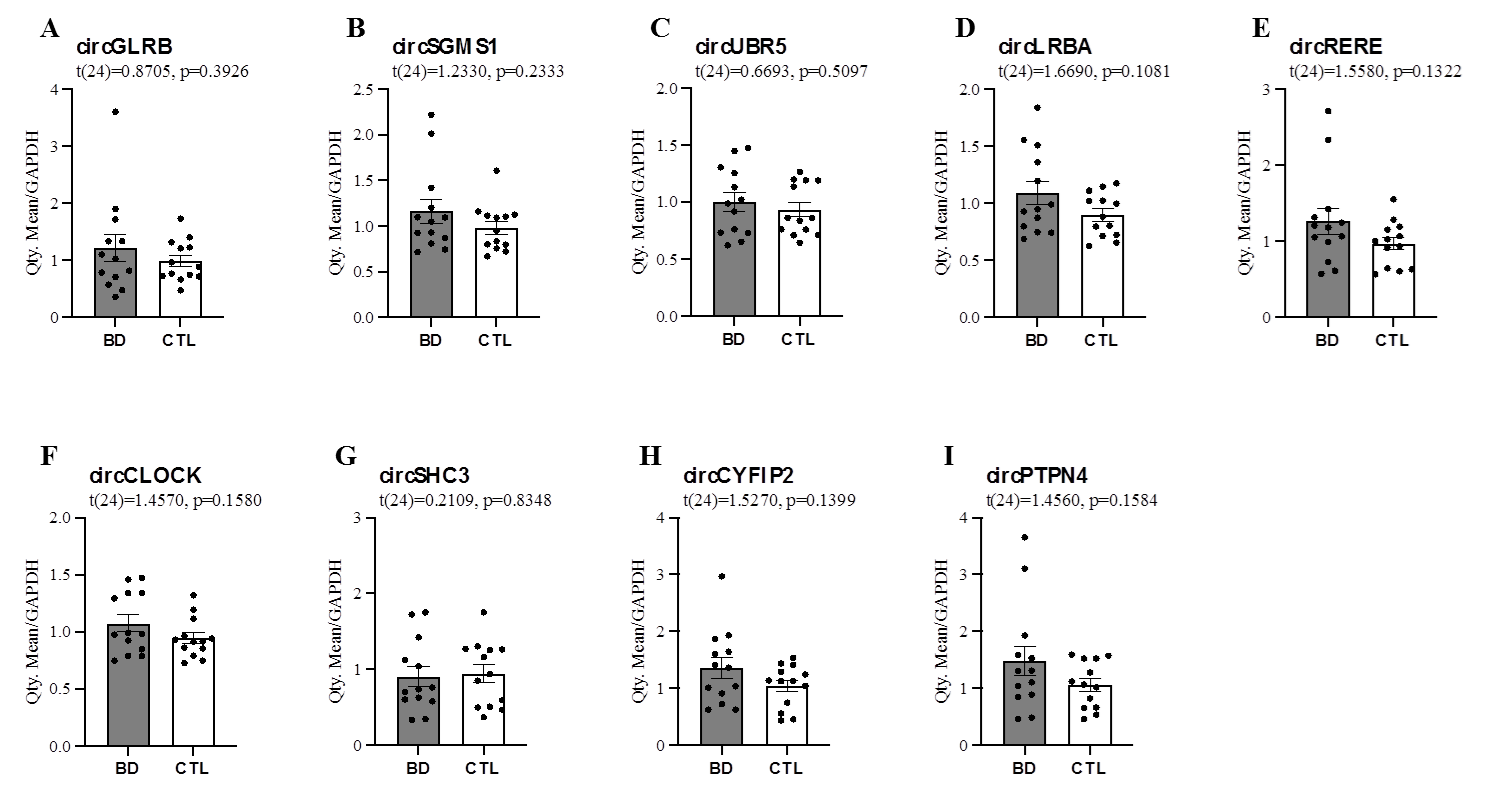
Supplementary Figure 2**

**Supplementary Figure 3**


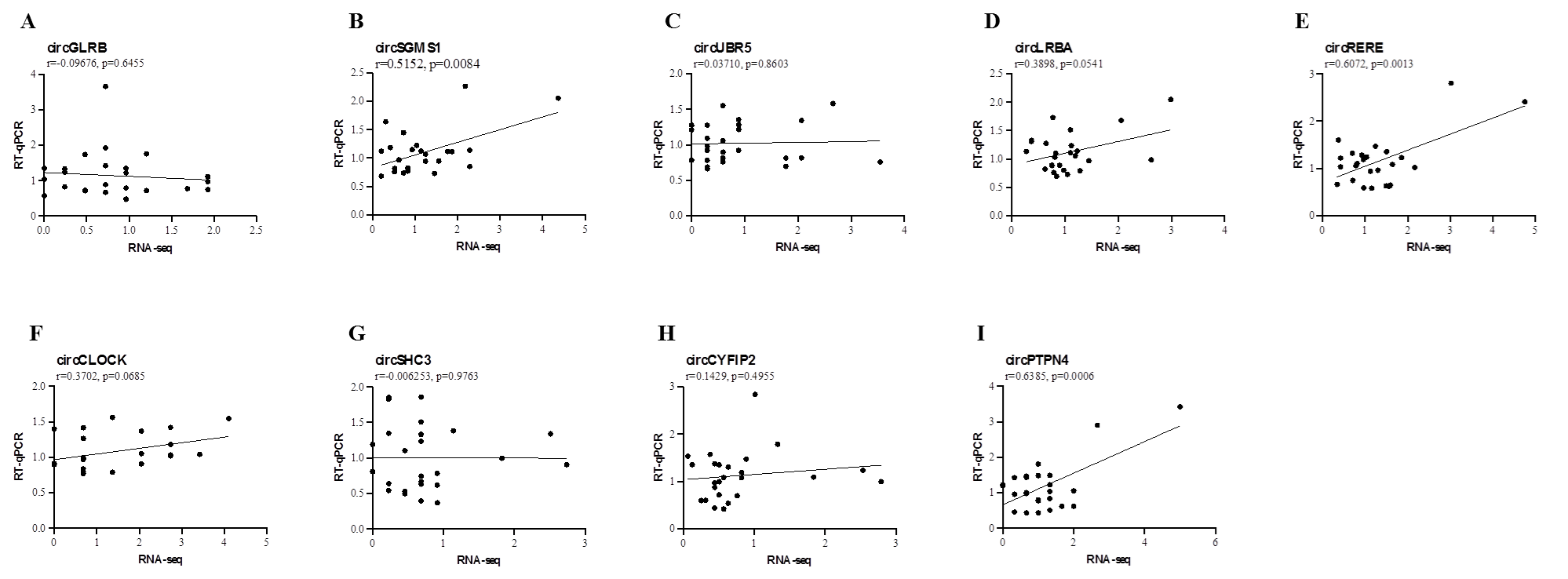


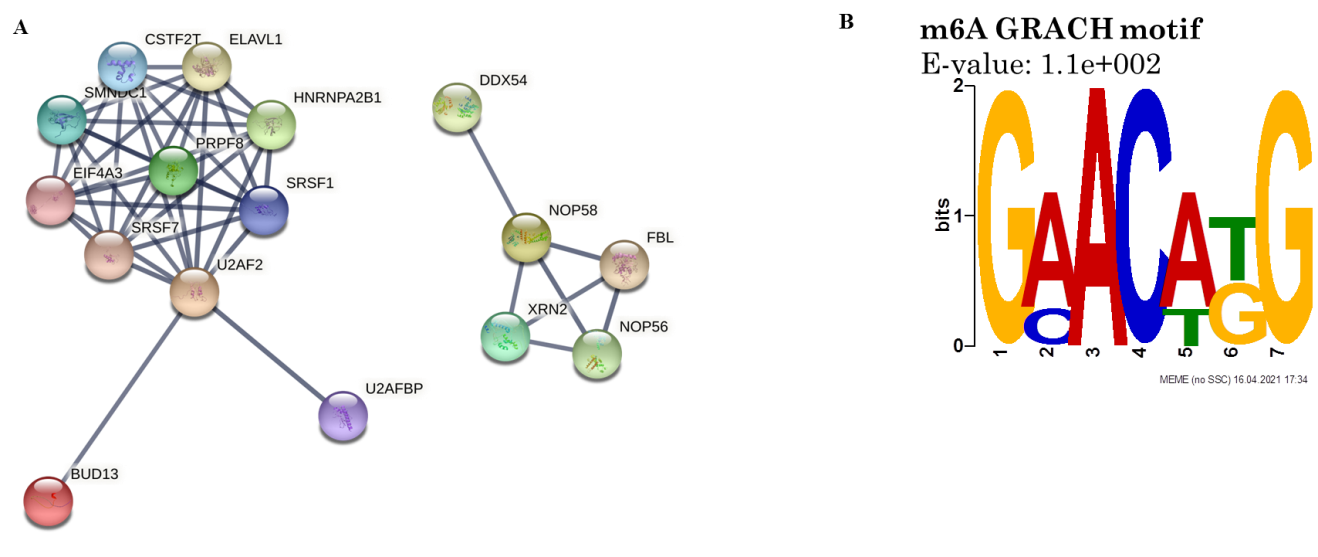
**Supplementary Figure 4**

**Supplementary Table 1**

| Primer | Sequence |
| --- | --- |
| CCNT2 fw | CCTCTAGAGCCACTGCTGGA |
| CCNT2 rev | TTGCTTGCTCTTACTAACTGGGT |
| 18S rRNA fw | CTCAACACGGGAAACCTCAC |
| 18S rRNA rev | CGCTCCACCAACTAAGAACG |
| GAPDH fw | TTGTCAAGCTCATTTCCTGG |
| GAPDH rev | TGTGAGGAGGGGAGATTCAG |
| circTulp4_fw | GGAGTGGTTGGGGTGACTTT |
| circTulp4_rev | TCAACTGCCATACGAAGCGT |
| circCCNT2 fw | TCACCATTGAACACCCACAC |
| circCCNT2 rev | CATTTAGTATCCAGCAGTGGCTCT |
| circPTPN4 fw | TTGGAGTGATGTCAGGAGGA |
| circPTPN4 rev | TCGGAAACGTGAGGTCATTA |
| circUBR5 fw | TACAGCCATTGCACAAGCAT |
| circUBR5 rev | ACCTGCTCGCAAACCACTAC |
| circSGMS1 fw | CAGCGACTGAAGGAGTGTG |
| circSGMS1 rev | CATTCTGGTCATCTTGGCTTC |
| circLRBA fw | TTGCCCTCAGTTCCAACAGT |
| circLRBA rev | TCAAATTCCGCATGTCTGTG |
| circRERE fw | GGGACCATCTCCTCATGAAC |
| circRERE rev | ATGAATCCACTCGGGCTTTA |
| circSHC3 fw | CTACAACAGCATCCCAAGCA |
| circSHC3 rev | TCTAGGTGACACAGGGCTGA |
| circCLOCK fw | TGTTATGTTGTTTACCGTAAGCTG |
| circCLOCK rev | GGTTTCCAGTCCTGTCGAAT |
| circCYFIP2 fw | TGTCTCCACCAGCAACTTGA |
| circCYFIP2 rev | ACAGCATGACCGCATACTCA |
| circGLRB fw | TGGCTTATTGCTTGCCTTCT |
| circGLRB rev | AAGGTCCTCTGCTGACTGCT |
| circCCNT2 TaqMan Probe | GTTAGTAAGAGATAATATCGTC |
